# Hidden by national averages: Unveiling the complex dynamics of ethnic disparities in under-five survival across sub-Saharan Africa

**DOI:** 10.1016/j.ssmph.2025.101870

**Published:** 2025-10-13

**Authors:** Vincent Bio Bediako

**Affiliations:** a Graduate Group in Demography, University of Pennsylvania, Philadelphia, USA; b International Max Planck Research School for Population, Health, and Data Science, Rostock, Germany; c Department of Population and Health, University of Cape Coast, Cape Coast, Ghana

**Keywords:** Under-five mortality, Ethnic disparities, Sub-Saharan Africa, Health inequalities, Socioeconomic factors, Maternal education, Decomposition analysis

## Abstract

Under-five mortality in sub-Saharan Africa has declined markedly, yet ethnic gaps persist and may even widen despite overall gains. This study uses two consecutive rounds of Demographic and Health Surveys (2007–2022) from twelve countries to trace how child characteristics, maternal education, household wealth, and urban-rural residence drive ethnic differentials in child survival. Country-specific total births ranged from 23,109 to 127,545 per survey round. Employing survey-weighted quasi-Poisson models with person-year offsets and a sequential Shapley-value decomposition, the study estimates crude and adjusted rate ratios for pairwise ethnic comparisons. It predicts mortality rates under counterfactual socioeconomic conditions. Results show enduring disparities, for example, among the Luo versus the Kalenjin in Kenya (adjusted RR 2.15, 95 % CI 1.83–2.51) and the Hausa versus the Yoruba in Nigeria (adjusted RR 1.39, 95 % CI 1.22–1.56), with maternal education accounting for roughly one-quarter to one-third of inequality. At the same time, the role of household wealth varies by context. Sensitivity analyses examining administrative boundary effects revealed that geographic mediation accounts for 15.4 % of ethnic disparities in Ghana's centralized system and 35.1 % in Nigeria's decentralized federal structure, suggesting that apparent ethnic disparities substantially reflect differential healthcare access across administrative units rather than intrinsic cultural factors. In Ghana, targeted policies combining fee removal and culturally tailored maternal care have noticeably narrowed ethnic gaps. These findings underscore that eliminating preventable child deaths by 2030 requires interventions that address both the socioeconomic and cultural determinants of ethnic disadvantage.

## 1 Introduction

Under-five mortality remains a key barometer of global health inequities, with the annual number of deaths falling by 51 percent since 2000, yet still amounting to 4.9 million in 2022 (UN IGME, 2023). Progress has been uneven: sub-Saharan Africa's under-five mortality rate of 74 per 1000 live births in 2021 was nearly double the global average, and stark within-country gaps persist along socioeconomic and ethnic lines (see Table 1, Table 2, Table 3).

**Table 1:** List of countries, sample size, and survey year.

Countries	Survey year 1	Sample size	Survey year 2	Sample year
Burkina Faso	2010	56178	2021	48745
Cameroon	2011	42312	2018	33988
DR. Congo	2007	29548	2014	59276
Gabon	2012	23109	2020	23232
Ghana	2014	23118	2022	34663
Guinea	2012	27683	2018	28887
Kenya	2014	83591	2022	77381
Nigeria	2013	119386	2018	127545
Senegal	2012	44928	2019	21562
Sierra Leone	2013	47392	2019	40543
Uganda	2011	28609	2016	57906
Zambia	2013	49207	2018	38446

**Table 2:** Variables used in the study.

Variables	Categories and code	Variable type
Dependent variable		
*Under-five mortality*		Categorical
	Dead = 1	
	Alive = 0	
Independent variable		
*Ethnicity*	Country specific	Categorical
Control variables		
*Educational level*		Categorical
	No formal schooling = 0	
	Primary schooling = 1	
	Secondary or above = 2	
*Type of residence*		Categorical
	Urban = 1	
	Rural = 2	
*Wealth status*		Categorical
	Poorest = 1	
	Poorer = 2	
	Middle = 3	
	Richer = 4	
	Richest = 5	
*Sex of child*		Categorical
	Male = 1	
	Female = 2	
*Child's age in months*		Continuous
*Survey year*	Country specific	Categorical

**Table 3:** Ethnic Disparities in crude and adjusted mortality rates.

Ethnicity	% share	crude rate (Survey 1)	Adjusted rate (Survey 1)	crude rate (Survey 2)	Adjusted rate (Survey 2)
**Eastern Africa**					
**Kenya**					
*Kalenjin*	17.8	1	1	1	1
*Luo*	13.2	2.17 (1.85–2.54)	2.15 (1.83–2.51)	2.15 (1.55–2.99)	2.13 (1.54–2.95)
*Kikuyu*	12.2	1.14 (0.94–1.39)	1.29 (1.06–1.56)	1.14 (0.79–1.63)	1.28 (0.89–1.83)
*Luhya*	11.8	1.51 (1.27–1.81)	1.56 (1.31–1.85)	1.50 (1.06–2.12)	1.55 (1.10–2.18)
*Somali*	8.8	1.33 (0.83–2.14)	1.49 (0.93–2.38)	1.32 (0.69–2.52)	1.48 (0.78–2.79)
*Kamba*	7.9	0.90 (0.73–1.12)	0.94 (0.76–1.17)	0.90 (0.61–1.31)	0.94 (0.64–1.37)
*Other*	7.5	1.48 (1.20–1.83)	1.44 (1.15–1.79)	1.47 (1.01–2.15)	1.43 (0.97–2.10)
*Mijikenda/Swahili*	5.4	1.18 (0.95–1.47)	1.14 (0.91–1.42)	1.18 (0.80–1.73)	1.13 (0.77–1.67)
*Meru*	5.3	1.18 (0.92–1.51)	1.23 (0.96–1.56)	1.18 (0.78–1.78)	1.22 (0.81–1.83)
*Kisii*	5.3	0.79 (0.61–1.03)	0.85 (0.65–1.09)	0.78 (0.51–1.21)	0.84 (0.55–1.28)
*Maasai*	2.7	0.68 (0.47–0.98)	0.64 (0.44–0.93)	0.68 (0.40–1.16)	0.64 (0.37–1.09)
*Taita/Taveta*	1.2	1.33 (0.83–2.14)	1.49 (0.93–2.38)	1.32 (0.69–2.52)	1.48 (0.78–2.79)
*Embu*	0.9	1.70 (0.92–3.13)	1.78 (0.97–3.25)	1.69 (0.78–3.69)	1.77 (0.82–3.81)
**Western Africa**					
**Nigeria**					
*Hausa*	32.4	1	1	1	1
*Other*	26.3	0.58 (0.53–0.63)	0.66 (0.60–0.71)	0.57 (0.49–0.67)	0.65 (0.57–0.75)
*Igbo*	12.0	0.49 (0.43–0.56)	0.76 (0.68–0.86)	0.49 (0.40–0.60)	0.76 (0.64–0.91)
*Yoruba*	10.5	0.41 (0.36–0.46)	0.72 (0.64–0.82)	0.40 (0.33–0.49)	0.72 (0.60–0.87)
*Fulani*	9.1	0.80 (0.71–0.89)	0.75 (0.67–0.83)	0.80 (0.66–0.96)	0.75 (0.63–0.88)
*Ijaw/Izon*	3.1	0.50 (0.40–0.62)	0.66 (0.55–0.81)	0.49 (0.37–0.66)	0.66 (0.51–0.85)
*Kanuri/Beriberi*	2.0	0.58 (0.47–0.72)	0.63 (0.52–0.77)	0.58 (0.43–0.77)	0.63 (0.49–0.81)
*Tiv*	1.8	0.48 (0.39–0.58)	0.49 (0.41–0.59)	0.47 (0.36–0.62)	0.49 (0.39–0.63)
*Ibibio*	1.5	0.34 (0.25–0.47)	0.50 (0.36–0.71)	0.34 (0.23–0.51)	0.50 (0.34–0.75)
*Igala*	0.9	0.49 (0.35–0.67)	0.61 (0.43–0.85)	0.48 (0.33–0.72)	0.61 (0.41–0.90)
*Ekoi*	0.3	0.66 (0.31–1.42)	0.84 (0.41–1.69)	0.66 (0.28–1.52)	0.84 (0.39–1.79)

**Central Africa**					
**Cameroon**					
*Arab-Choa*	35.2	1	1	1	1
*Other*	30.3	0.75 (0.66–0.86)	0.89 (0.78–1.01)	0.74 (0.58–0.95)	0.88 (0.69–1.11)
*Biu-Mandara*	23.7	0.69 (0.61–0.78)	1.04 (0.92–1.18)	0.68 (0.54–0.87)	1.03 (0.81–1.30)
*Grassfields*	4.0	0.35 (0.26–0.48)	0.56 (0.42–0.76)	0.35 (0.22–0.53)	0.56 (0.37–0.84)
*Kako/Meka/Pygmé*	2.4	1.08 (0.78–1.48)	1.28 (0.95–1.71)	1.06 (0.69–1.65)	1.26 (0.84–1.89)
*Bantoïde Southwest*	1.3	0.95 (0.63–1.42)	0.81 (0.53–1.23)	0.94 (0.55–1.58)	0.80 (0.47–1.36)
*Adamaoua-Oubangui*	1.2	2.24 (1.66–3.02)	1.82 (1.39–2.39)	2.22 (1.46–3.36)	1.80 (1.23–2.64)
*Bamilike/Bamoun*	1.0	0.63 (0.30–1.33)	0.87 (0.42–1.80)	0.62 (0.26–1.48)	0.86 (0.38–1.99)
*Beti/Bassa/Mbam*	0.9	0.91 (0.58–1.42)	1.26 (0.79–2.00)	0.90 (0.51–1.58)	1.25 (0.70–2.21)
**Southern Africa**					
**Zambia**					
*Bemba*	31.2	1	1	1	1
*Other*	30.2	1.03 (0.90–1.16)	1.03 (0.91–1.16)	1.03 (0.79–1.33)	1.03 (0.80–1.32)
*Tonga*	12.6	0.78 (0.63–0.95)	0.82 (0.67–1.01)	0.78 (0.56–1.09)	0.82 (0.59–1.15)
*Nyanja*	9.0	0.91 (0.75–1.09)	0.90 (0.74–1.08)	0.91 (0.66–1.25)	0.90 (0.65–1.23)
*Lozi*	6.5	1.04 (0.80–1.34)	1.01 (0.79–1.30)	1.04 (0.70–1.53)	1.01 (0.69–1.48)
*Lunda*	3.8	0.61 (0.46–0.80)	0.61 (0.47-0.80	0.61 (0.40–0.91)	0.61 (0.41–0.91)
*Kaonde*	3.6	0.70 (0.51–0.95)	0.72 (0.53–0.99)	0.70 (0.45–1.08)	0.72 (0.47–1.12)
*Luvale*	3.1	0.70 (0.45–1.09)	0.68 (0.44–1.07)	0.71 (0.40–1.25)	0.68 (0.39–1.21)

Building on the Mosley–Chen analytical framework, which situates child survival as the product of social, economic, and biological determinants operating through proximate mechanisms (e.g., nutrition, access to care) (Black et al., 2003), this study also draws on equity-oriented approaches to health inequality. Prior work documents pronounced ethnic differentials often exceeding one-third higher mortality among politically marginalized groups in multiple sub-Saharan settings (Brockerhoff & Hewett, 2000).

While previous studies have documented ethnic health disparities, a critical gap remains in understanding whether these disparities reflect cultural differences or geographic-institutional factors. Administrative boundaries structure healthcare delivery, resource allocation, and policy implementation in sub-Saharan Africa, yet their mediating role in ethnic health disparities remains unexplored. This distinction is crucial: if ethnic disparities primarily operate through administrative geography, place-based rather than ethnicity-targeted interventions may prove more effective.

This paper exploits Demographic and Health Surveys from twelve countries to decompose changes in under-five mortality gaps between majority and minority ethnic groups across two survey waves. Using survey-weighted quasi-Poisson regression with person-year offsets and Shapley-value decomposition, we quantify the shifting contributions of child, maternal, and household factors to ethnic disparities. Our findings reveal marked convergence in Ghana, widening gaps in Zambia, and heterogeneous patterns elsewhere.

## 2 Background

Under-five mortality in sub-Saharan Africa has declined dramatically from 93 deaths per 1000 live births in 1990 to 37 per 1000 in 2020, yet this aggregate progress conceals persistent, and in some settings widening, gaps between ethnic groups (Victora et al., 2020; Antai, 2011). Ethnicity in this region reflects cultural practices such as breastfeeding norms and health-seeking behaviors and mirrors historical patterns of infrastructure investment and political power that shape access to care (Luke, 2023). For example, Nigeria's north-south divide aligns closely with Hausa versus Yoruba territories, producing entrenched differences in facility density and child survival outcomes (Adedini et al., 2023), while in Ethiopia, traditional feeding customs persist even among urban, educated households, directly affecting child health (Moreda et al., 2025).

Although socioeconomic inequalities in health by wealth, maternal education, and urban-rural residence have been well documented (Houweling et al., 2003), fewer studies have systematically unpacked the mechanisms driving ethnic disparities. Brockerhoff and Hewett (2000) first demonstrated across eleven African countries that some ethnic groups enjoy consistently lower under-five mortality. Victora et al. (2020) extended this analysis to 36 low- and middle-income nations. Little effort has been devoted to decomposing how child characteristics, maternal education, household wealth, and residence contribute to these gaps. Studies have not examined how crude and adjusted rate ratios evolve across successive survey rounds.

The intersection of ethnicity and administrative geography presents a methodological challenge in disentangling cultural from institutional determinants of health disparities. Colonial administrative structures often reinforced ethnic boundaries, creating spatially clustered disadvantage that persists through contemporary healthcare systems (Mamdani, 1996). Recent evidence from decentralized health systems suggests that subnational variation in healthcare quality may explain substantial portions of ethnic health gaps (Bossert et al., 2003; Okoli et al., 2020). However, no study has systematically quantified the mediating role of administrative boundaries in ethnic under-five mortality disparities across diverse African health system structures.

This study fills these gaps using Demographic and Health Surveys from twelve sub-Saharan countries. Employing survey-weighted quasi-Poisson models with person-year offsets and Shapley-value decomposition, the study traces how maternal (education, age) and household (wealth, residence) factors jointly shape ethnic disparities in under-five mortality. The study compares crude and adjusted rate ratios across two DHS waves to reveal divergent trajectories, such as Ghana's pronounced convergence versus Burkina Faso's persistent Fulfuldé/Peul disadvantage, and conducts pairwise analyses to uncover nuanced patterns beyond majority minority contrasts. By elucidating these mechanisms, our findings offer targeted insights for policymakers aiming to “leave no one behind” in the Sustainable Development Goals' drive to end preventable child deaths by 2030.

## 3 Methods

### 3.1 Data sources and study design

This study analyzes data from the two most recent Demographic and Health Surveys (DHS) for twelve Sub-Saharan African countries from 2007 to 2022. The DHS datasets were selected for their standardized and validated methodologies, enabling robust cross-country comparisons (Fabic et al., 2012; Rutstein & Rojas, 2006). Recent advancements in demographic modeling, particularly the Bayesian B-spline and data-integration approaches, have enhanced the accuracy of under-five mortality estimates (Alkema & New, 2014; Hill et al., 2012), informed the selection of countries, prioritizing those meeting the Inter-agency Group for Under-five Mortality Estimation (UN IGME) criteria, and provided ethnicity-disaggregated data.

The DHS employs a stratified two-stage cluster sampling design, first selecting those primary clusters that regions and urban/rural status stratify, then interviewing the households from within the clusters, researchers revalidated the whole design for reliability in resource-limited settings (see Optimal Sample Sizes for Two-stage Cluster Sampling in DHS; WP30 Working Paper, DHS Program) (DHS Program, 2010, DHS Program, 2012). To guarantee statistical strength and representativeness, we kept ethnic groups with at least 500 live births per survey. This threshold aligns with standard DHS subgroup analysis guidelines and optimal variance considerations.

### 3.2 Study population and sample selection

The analytical sample included children aged 0–59 months, drawn from the DHS birth recording files. Country-specific total births ranged from 23,109 to 127,545 per survey round. Birth histories provided precise survival time measures for deceased and surviving children. To balance recall bias with sample size adequacy, the analysis was restricted to children born within 10 years preceding each survey.

## 4 Variables

### 4.1 Outcome variable

Under-five mortality was defined as death before the fifth birthday. Exposure time was calculated from birth to the date of death for deceased children or the survey date for surviving children. Age at death was categorized into days (neonatal deaths), months (post-neonatal deaths), and years (child deaths) for precision.

### 4.2 Control variables

Variables included child age (following WHO survival analysis guidelines), sex, maternal education, household wealth, and residence type. These variables align with theoretical frameworks on the social determinants of health (Solar & Irwin, 2010) and empirical evidence on under-five mortality in Sub-Saharan Africa (Balaj et al., 2021).

### 4.3 Independent variable

Ethnicity was operationalized based on self-reported ethnic group identification from the DHS surveys. For statistical robustness and to ensure reliable estimates, the analysis was restricted to ethnic groups with more than 500 live births within each country. The ethnic classification follows country-specific groupings recorded in the DHS, which captures the major ethnic divisions within each national context. For example, Burkina Faso included the Mossi (reference group), Fulfuldé/Peul, Gourmatche, and other ethnic categories. In Nigeria, the classification included major groups such as the Hausa (reference group), Yoruba, Igbo, and other ethnic identities. This approach to ethnic classification maintains consistency with national demographic categories while ensuring sufficient sample sizes for statistical analysis.

For each country's analysis, the largest ethnic group was typically used as the reference category for comparative studies. This methodological choice facilitates the interpretation of relative disparities while acknowledging the demographic composition of each nation. However, it's important to note that being the largest group does not necessarily correlate with socioeconomic advantages or better health outcomes, as demonstrated in the results.

## 5 Analytical approach

### 5.1 Person-years calculation

The study adopted a person-years framework to account for varying exposure times in mortality risk, consistent with demographic methods (Preston et al., 2001). For:

• Surviving children: exposure was calculated from birth to the survey date.
• Deceased children: exposure was from birth to the date of death.
• Exposure was from the truncation date to the date of death or survey for children born before the truncation date.

A 10-year recall period minimized recall bias while maintaining ethnic-specific sample sizes.

### 5.2 Survey design implementation

To address DHS's complex sampling design, survey design objects were constructed using the svydesign() function in R, incorporating clustering at the primary sampling unit (v001), stratification by geographic region (v024) and residence type (v025), and sampling weights for national representativeness.

## 6 Statistical modeling strategy

### 6.1 Base model

Ethnic disparities in mortality rates were assessed using survey-weighted generalized linear models (GLMs) with a quasipoisson family to handle overdispersion. The study employed survey-weighted generalized linear models instead of survival analysis techniques for several theoretical plus practical reasons, using quasi-Poisson GLMs. GLMs with log-link functions and person-year offsets are mathematically equivalent initially to piecewise exponential survival models. Those models assume constant hazards within age intervals (Breslow, 1974; Rodriguez, 2007). This equivalence ensures that the analytical approach rigorously analyzes survival and offers computational advantages. The base model included ethnicity, survey year, and their interaction:

log(μᵢ) = log(PYᵢ) + β_0_ + β_1_Ethnicityᵢ + β_2_Survey-yearᵢ + β_3_(Ethnicity × Survey-year)ᵢ

Where μᵢ represents expected deaths, PYᵢ is person-years exposure (offset), and the ethnicity-year interaction captures temporal changes in disparities.

### 6.2 Adjusted model

Socioeconomic covariates (maternal education, wealth, and residence) were incorporated to examine how these factors mediated ethnic disparities:

log(μᵢ) = log(PYᵢ) + β_0_ + β_1_Ethnicityᵢ + β_2_Survey-yearᵢ + β_3_(Ethnicity × Survey-year) ᵢ + β_4_Ageᵢ + β_5_Sexᵢ + β_6_Educationᵢ + β_7_Wealthᵢ + β_8_Residenceᵢ

This specification assessed how socioeconomic factors mediate ethnic disparities in under-five mortality.

## 7 Statistical analysis and rate estimation

### 7.1 Mortality rate estimation framework

The analysis employed an appropriate approach to estimating ethnic-specific mortality rates and their temporal evolution. The estimation process proceeded through several carefully specified stages:

First, model coefficients associated with ethnic group effects were extracted from the generalized linear models to quantify baseline disparities. These coefficients were transformed through exponentiation to obtain interpretable rate ratios, directly comparing mortality risks between ethnic groups. Confidence intervals were calculated using the model parameters' full variance-covariance matrix, ensuring proper uncertainty accounting in both main effects and interactions.

The baseline model was specified as:

Rate = exp(β_0_ + β_e_ + βᵧ + β_e_ᵧ)

Where:

β_0_ represents the intercept

β_e_ represents the ethnic group effect

βᵧ represents the year effect

β_e_ᵧ represents the ethnicity-by-year interaction.

### 7.2 Prediction and visualization of mortality rates

A standardized prediction dataset was constructed to generate comparable mortality predictions across ethnic groups. We specifically made a grid that included every combination of ethnic groups. The grid also had all of the survey years within it. We set person-years of exposure to 1.0 (unity) for each combination, allowing predicted values to represent annual mortality rates per person-year rather than counts.

For covariate standardization, we held all variables at reference values, including child age at 12 months, male sex, no maternal education, poorest wealth quintile, and rural residence. This approach affects mortality by isolating the “pure” ethnic effect when these groups do not differ in composition. Standardized data did not refit the models. Predictions instead under these standardized conditions were generated using the original model coefficients.

Predicted rates were computed using fitted models, incorporating point estimates and uncertainty. For each ethnic-period combination, we calculated mortality rates per 1000 person-years with corresponding 95 % confidence intervals derived from the standard errors of the linear predictors. The confidence intervals were computed as exp(fit ± 1·96 × SE) × 1000, where SE represents the standard error of prediction on the link scale.

The results were visualized using forest plots, displaying both crude and adjusted rates with their confidence intervals for each ethnic group. The plots were stratified by model type (crude versus adjusted) to facilitate a comparison of ethnic disparities before and after socioeconomic adjustment. Ethnic groups were ordered by mortality rate magnitude to highlight the hierarchy of risk, with separate estimates shown for each survey period to illustrate temporal changes in disparities.

### 7.3 Pairwise comparisons and temporal analysis

To compare ethnic pairs for each country, the study employed estimated marginal means to conduct pairwise comparisons between ethnic groups stratified by survey year. This stratification examined how ethnic disparities evolved while accounting for the complex survey design. The contrasts were computed on the log scale from the generalized linear models and subsequently transformed to obtain rate ratios and their corresponding confidence intervals.

For each survey year, the analysis generated a complete set of pairwise comparisons between ethnic groups, with significance levels denoted using conventional thresholds (p < 0.001: ∗∗∗, p < 0.01: ∗∗, p < 0.05: ∗). This approach provided a detailed assessment of which ethnic differences in mortality rates were statistically significant while controlling for multiple comparisons to maintain appropriate family-wise error rates.

### 7.4 Decomposition analysis

The study employed a sequential decomposition approach to understand the pathways through which ethnic disparities in under-five mortality operate. This methodological choice was grounded in the theoretical framework Mosley and Chen (1984) developed for analyzing child survival in developing countries, which posits that socioeconomic determinants operate through proximate determinants to influence under-five mortality. The sequential model-building strategy enables quantification of how different socioeconomic factors mediate the relationship between ethnicity and under-five mortality.

The decomposition proceeded through five carefully specified models:

#### 7.4.1 Model 1 (base model)

log(μᵢ) = log(PYᵢ) + β_0_ + β_1_Ethnicityᵢ + β_2_Yearᵢ + β_3_(Ethnicity × Year)ᵢ

This baseline model established the total ethnic disparities in mortality before accounting for any mediating factors. This model's ethnicity coefficients (β_1_) represent the “total” ethnic effect on mortality.

#### 7.4.2 Model 2 (child characteristics)

log(μᵢ) = Model 1 + β_4_Ageᵢ + β_5_Sexᵢ

The addition of child demographic characteristics accounts for compositional differences in age and sex distribution across ethnic groups. Changes in ethnic coefficients between Models 1 and 2 represent the portion of ethnic disparities attributable to these fundamental demographic factors.

#### 7.4.3 Model 3 (maternal education)

log(μᵢ) = Model 2 + β_6_Educationᵢ

Incorporating maternal education captures one of the key pathways through which ethnic disparities may operate. Educational differences often reflect historical and structural inequalities in access to schooling across ethnic groups. The change in ethnic coefficients between Models 2 and 3 quantifies the contribution of educational disparities to ethnic differences in under-five mortality.

#### 7.4.4 Model 4 (household wealth)

log(μᵢ) = Model 3 + β_7_Wealthᵢ

Adding household wealth accounts for economic resources available to families, another crucial pathway through which ethnic disparities may manifest. Changes in ethnic coefficients between Models 3 and 4 represent the portion of ethnic disparities attributable to wealth inequalities.

#### 7.4.5 Model 5 (full model)

log(μᵢ) = Model 4 + β_8_Residenceᵢ

The final model incorporates urban/rural residence, capturing geographic aspects of inequality that may contribute to ethnic disparities in under-five mortality. Changes in coefficients between Models 4 and 5 represent the contribution of residential patterns to ethnic mortality differentials.

For each step in the decomposition, the contribution of the added factor(s) was calculated as:

Contribution_k_ = [(β_ek-1_ - β_ek_)/β_e1_] × 100

Where:

β_ek-1_ is the ethnic coefficient from the previous model

β_ek_ is the ethnic coefficient from the current model

β_e1_ is the ethnic coefficient from the base model.

This sequential approach offers several methodological advantages:

a. It enables the identification of the relative importance of different pathways through which ethnic disparities operate, informing potential policy interventions.
b. The stepwise inclusion of variables allows for assessing how different socioeconomic factors interact and potentially compound or mitigate ethnic disparities.
c. Any remaining ethnic disparities after full adjustment (residual inequality) suggest the presence of other unmeasured factors or direct effects of ethnicity on under-five mortality.

The method provides insights into the magnitude and mechanisms of health disparities, moving beyond simple documentation of inequalities to understanding their sources.

However, several methodological considerations warrant attention. First, the order of variable inclusion can affect the estimated contributions of different factors. Second, the approach assumes that the relationship between ethnicity and covariates remains stable across models. Third, unmeasured confounding may affect the estimated contributions of included variables. These limitations were carefully considered when interpreting the decomposition results.

This decomposition strategy provides valuable insights into the mechanisms generating ethnic disparities in under-five mortality, helping to identify potential intervention points for reducing health inequalities. The results can inform policy by highlighting whether disparities primarily operate through educational differences, economic inequalities, geographic factors, or other pathways.

### 7.5 Sensitivity analysis: geographic mediation effects

To assess the robustness of findings to alternative specifications of geographic context, we conducted a sensitivity analysis examining administrative boundary mediation of ethnic disparities using Ghana and Nigeria as comparative case studies. This analysis addresses potential confounding by geographic factors beyond rural-urban classification and tests whether apparent ethnic disparities reflect cultural differences or differential exposure to administrative-institutional contexts. The result of the sensitivity analysis is presented in Appendix 3.

### 7.6 Case selection and design

The selected Ghana and Nigeria to provide contrasting healthcare system structures: Ghana's centralized National Health Insurance Scheme versus Nigeria's decentralized federal health governance. Ghana's DHS surveys (2014, 2022) offered a unique analytical opportunity, as six new administrative regions were created between surveys, enabling examination of how boundary changes intersect with ethnic distributions. Nigeria's surveys (2013, 2018) complemented this by testing geographic mediation effects in a federal system with state-level healthcare autonomy.

### 7.7 Geographic classifications

***Administrative Boundary Harmonization***: For Ghana, we harmonized the 2014 ten-region structure with the 2022 sixteen-region structure by mapping new regions (Ahafo, Bono East, North East, Oti, Savannah, Western North) to their parent regions while preserving analytical capacity to test ethnicity-region interactions.

***Homeland-Diaspora Classification***: We classified ethnic residence as “homeland” (traditional territory) versus “diaspora” (non-traditional areas) based on documented historical settlement patterns and linguistic distributions. Classifications included: Ghana - Akan (Ashanti, Central, Western, Eastern, Bono regions), Ga/Dangme (Greater Accra), Ewe (Volta, Oti), Mole-Dagbani (Northern, Savannah, North East); Nigeria - Hausa-Fulani (northern states), Yoruba (southwest states), Igbo (southeast states), with other groups classified using colonial administrative records.

### 7.8 Sequential mediation models

We estimated four model specifications to quantify geographic mediation. The sequential approach quantifies the proportion of apparent ethnic disparities attributable to administrative boundary effects, testing whether ethnic disparities persist after controlling for geographic-institutional contexts.

### 7.9 Model specifications

Extended models incorporated administrative boundaries and homeland interactions:

log(μᵢ) = β_0_ + β_1_Ethnicityᵢ + β_2_AdminRegionᵢ + β_3_HomelandStatusᵢ + β_4_(Ethnicity × AdminRegion)ᵢ + β_5_(Ethnicity × Homeland)ᵢ + β_6_Xᵢ + εᵢ

Where μᵢ represents under-five mortality risk, AdminRegionᵢ captures administrative boundary effects, HomelandStatusᵢ indicates traditional versus non-traditional residence, and Xᵢ includes standard demographic and socioeconomic controls.

### 7.10 Analytical rationale

This sensitivity analysis addresses three methodological concerns: (1) geographic confounding beyond rural-urban differences, (2) policy implementation through administrative rather than ethnic frameworks, and (3) differential risks for ethnic groups residing outside traditional territories. The comparative design assesses whether geographic mediation varies by healthcare system structure, providing external validation across different institutional contexts.

Model performance was evaluated using likelihood ratio tests for nested comparisons and information criteria for assessing ethnicity-region interaction effects. Cross-system consistency and temporal stability across survey years provide robustness checks for geographic mediation patterns.

### 7.11 Validation and diagnostics

Model diagnostics included assessment of deviance residuals, interaction testing via likelihood ratio tests, and evaluation of overdispersion assumptions. Sensitivity analyses addressed alternative ethnic classifications and truncation dates.

### 7.12 Data limitations

First, depending on the ethnicity reported to DHS, it can hide identities that change because it could misclassify people in comparisons over time. Second, the DHS has no direct indicators to show healthcare quality, such as facility readiness or provider competence, limiting our ability to capture intra-country variation in service delivery. Third, the study made adjustments for each child, including maternal factors and household factors, but residual confounding may persist. Varied cultural customs differ among ethnic groups, influencing child survival, like dietary taboos, postpartum seclusion, or reliance on healers.

Classifiers of homelands draw on how settlers historically settled per colonial records and linguistic surveys, though they may not reflect recent internal migrations or multiple ethnic affiliations. Nonetheless, it systematically approaches testing how geography mediates ethnic disparities. Administrative boundaries follow official classifications for when each survey occurs, and harmonization ensures comparability concerning time. These boundaries are assumed to proxy for differences in healthcare provision, resource allocation, and policy implementation. Observed regional contrasts across Ghana and Nigeria in health indicators support this assumption.

## 8 Results

### 8.1 Ethnic disparities in crude and adjusted rate ratios

Across the twelve Sub-Saharan African countries studied, substantial ethnic disparities in under-5 mortality persisted between survey periods, with both crude and adjusted analyses revealing significant variations in mortality risk. Given the expected length of the results table, we present results from one country per sub-region of sub-Saharan Africa in the main table, with the full results provided in the supplementary materials. The estimated rates are shown in the table below:

#### 8.1.1 Eastern Africa

In Kenya, substantial ethnic disparities emerged in both crude and adjusted analyses. The Luo exhibited significantly higher mortality compared to the Kalenjin reference group in both crude (RR 2·17, 95 % CI 1·85-2·54) and adjusted analyses (RR 2·15, 95 % CI 1·83-2·51). These disparities persisted in the second survey period (adjusted RR 2·13, 95 % CI 1·54-2·95). The Luhya also showed elevated mortality (adjusted RR 1·56, 95 % CI 1·31-1·85), while the Maasai demonstrated lower mortality (adjusted RR 0·64, 95 % CI 0·44-0·93). In Uganda, the Baganda reference group showed intermediate mortality levels, with the Banyankole displaying elevated mortality (adjusted RR 1·30, 95 % CI 1·06-1·60). The Iteso demonstrated significantly lower mortality (adjusted RR 0·70, 95 % CI 0·56-0·88), while the Bafumbira showed a higher risk (adjusted RR 1·37, 95 % CI 1·00–1·88).

#### 8.1.2 Western Africa

Nigerian patterns revealed the Yoruba's substantially lower mortality compared to the Hausa reference group in both crude (RR 0·41, 95 % CI 0·36-0·46) and adjusted analyses (RR 0·72, 95 % CI 0·64-0·82). The Igbo showed similar protective effects (adjusted RR 0·76, 95 % CI 0·68-0·86). Other ethnic groups, including the Ijaw/Izon (adjusted RR 0·66, 95 % CI 0·55-0·81) and Kanuri/Beriberi (adjusted RR 0·63, 95 % CI 0·52-0·77), also demonstrated lower mortality than the Hausa. In Burkina Faso, using the Mossi as a reference, the Gourmantche showed significantly higher mortality (adjusted RR 1·37, 95 % CI 1·18-1·58), while the Bissa demonstrated lower mortality (adjusted RR 0·61, 95 % CI 0·50-0·74). The Senoufo maintained elevated mortality (adjusted RR 1·52, 95 % CI 1·33-1·74) across both surveys. In Guinea, the Malinké showed higher mortality compared to the Peulh reference group (adjusted RR 1·20, 95 % CI 1·05-1·37), while the Guerzé demonstrated lower mortality (adjusted RR 0·70, 95 % CI 0·51-0·95).

#### 8.1.3 Central Africa

In Cameroon, using the Arab-Choa as a reference, the Adamaoua-Oubangui showed significantly higher mortality (adjusted RR 1·82, 95 % CI 1·39-2·39), and the Grassfields demonstrated lower mortality (adjusted RR 0·56, 95 % CI 0·42-0·76). The Biu-Mandara showed varying patterns before and after adjustment (crude RR 0·69 to adjusted RR 1·04). The Democratic Republic of Congo revealed more modest ethnic variations, with the Uele Lake Albert group showing elevated mortality (adjusted RR 1·21, 95 % CI 0·90-1·62) compared to the Kasai, Katanga, and Tanganika reference group.

### 8.2 Southern Africa

In Zambia, using the Bemba as a reference, the Tonga showed lower mortality (adjusted RR 0·82, 95 % CI 0·67-1·01), while the Lunda demonstrated significantly lower mortality (adjusted RR 0·61, 95 % CI 0·47-0·80).

### 8.3 Pairwise comparisons between ethnic groups

The pairwise comparisons between ethnic groups revealed significant mortality differentials across the twelve Sub-Saharan African countries, with several countries showing persistent contrasts between survey periods. These comparisons, adjusted for multiple testing using Tukey's method, demonstrated stable and evolving patterns of ethnic disparities in child survival. The detailed results of these pairwise contrasts are presented in Appendix 1.

#### 8.3.1 Eastern African contrasts

Kenya's pairwise comparisons revealed persistent differences between ethnic groups. The Luo-Kalenjin contrast remained significant across surveys (2014: RR = −0·773, p < 0·001; 2022: RR = −0·754, p < 0·001). Significant contrasts also emerged between the Luo and Kikuyu (RR = 0·575, p < 0·001), Luo and Kamba (RR = 0·887, p < 0·001), and Luhya-Kisii (RR = 0·709, p < 0·001). In Uganda, significant contrasts emerged between the Iteso and Banyankole (RR = −0·444, p < 0·01) and between the Banyankole and Bagisu (RR = 0·387, p < 0·05).

#### 8.3.2 Western African comparisons

Nigeria's pairwise comparisons revealed significant contrasts between multiple ethnic pairs. The Hausa-Yoruba contrast showed prominence (RR = 1·027, p < 0·001 in 2018), while the Hausa-Igbo contrast maintained significance (RR = 0·761, p < 0·001). The Yoruba-Fulani comparison revealed substantial differences (RR -0·800, p < 0·001).

In Burkina Faso, significant contrasts emerged between the Mossi and Gourmantche (RR = −0·306, p < 0·01), Mossi and Bissa (RR = 0·473, p < 0·001), and Bissa-Senoufo (RR = −0·887, p < 0·001).

Guinea showed significant contrasts between the Malinké and Soussou (RR = 0·320, p < 0·05) and between Malinké and Guerzé (RR = 0·533, p < 0·01).

#### 8.3.3 Central and Southern African comparisons

In Cameroon, significant contrasts emerged between the Arab-Choa and Adamaoua-Oubangui groups (RR = −0·611, p < 0·001). The Grassfields showed substantial differences with multiple groups, particularly with the Adamaoua-Oubangui (RR = −1·180, p < 0·001). Zambia's analysis revealed significant contrasts between the Bemba and Lunda (RR = 0·533, p < 0·05) and between Other ethnic groups and Lunda (RR = 0·566, p < 0·01).

### 8.4 Predicted mortality rates

**Fig. 1:**
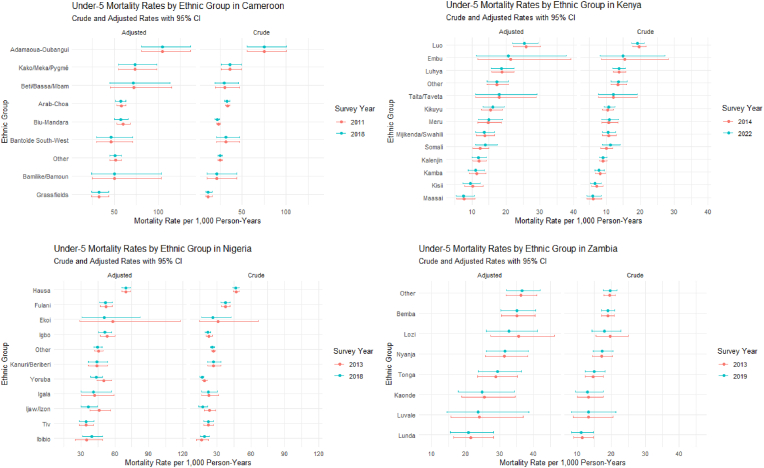
The predicted mortality rates for selected countries.

To examine ethnic-specific mortality patterns while accounting for socioeconomic factors, we calculated predicted under-five mortality rates for each ethnic group, holding covariates constant at reference values. These predictions reveal substantial variations in mortality risk across ethnic groups, even after standardizing maternal education, household wealth, and residence type. The predicted rates and their 95 % confidence intervals are presented in Fig. 1.

#### 8.4.1 Eastern Africa

Predicted mortality rates in Kenya showed that the Luo maintained consistently higher mortality across both survey periods. After adjustment for socioeconomic factors, predicted rates remained more than twice those of the Kalenjin reference group. The Maasai demonstrated notably lower predicted rates, while the Luhya maintained intermediate positions. In Uganda, predicted rates showed more nuanced patterns, with the Baganda reference group showing intermediate mortality levels. The Banyankole and Bakiga displayed elevated rates that persisted after adjustment, while the Iteso demonstrated consistently lower predicted mortality.

#### 8.4.2 Western Africa

Nigerian predictions revealed clear mortality hierarchies, with the Hausa showing substantially higher rates than other groups. The Yoruba and Igbo demonstrated markedly lower predicted mortality in crude and adjusted analyses, though adjustment attenuated these differences. Burkina Faso's predictions showed that Mossi had intermediate mortality levels, while the Gourmantche and Senoufo displayed significantly higher predicted rates. The Bissa consistently demonstrated lower mortality, with these patterns persisting after socioeconomic adjustment. Guinea's predicted rates showed the Malinké with elevated mortality compared to the Peulh reference group, while the Toma demonstrated significantly lower predicted rates.

#### 8.4.3 Central and Southern Africa

Cameroon's predicted rates showed the Adamaoua-Oubangui group had substantially higher mortality than others, while the Grassfields group demonstrated notably lower predicted mortality. These differences remained significant after adjustment.

In Zambia, predicted rates showed the Bemba with intermediate mortality levels, while the Lunda and Kaonde demonstrated consistently lower predicted mortality rates across both survey periods.

### 8.5 Decomposition analysis

**Fig. 2:**
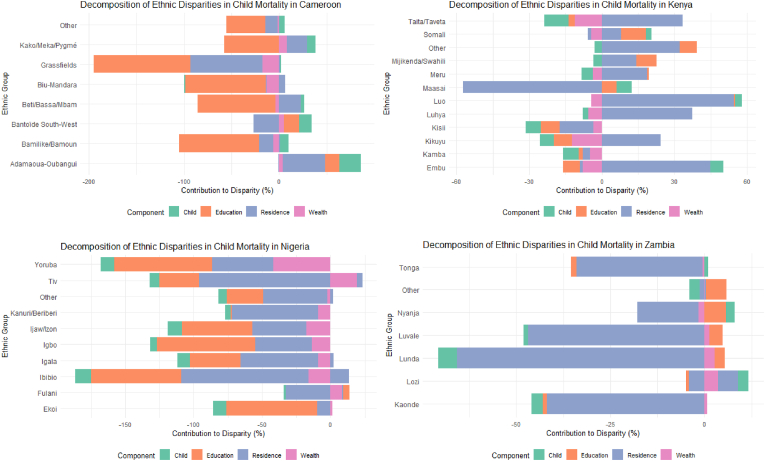
Decomposition analysis of the relative contribution of SES to ethnic mortality disparities in selected countries.

The sequential decomposition analysis revealed varying contributions of different factors to ethnic disparities in under-five mortality across countries. Through progressive model building, from child characteristics to socioeconomic factors, we quantified how each component contributed to observed ethnic differences in mortality risk. The results of this decomposition analysis are presented in Fig. 2, showing the relative contribution of each factor to ethnic mortality disparities.

#### 8.5.1 Eastern Africa

In Kenya, decomposition revealed that child characteristics explained 15–20 % of observed ethnic disparities. Maternal education contributed substantially to the Luo-Kalenjin disparity (approximately 25 %). After accounting for all measured factors, about 40 % of this disparity remained unexplained. Uganda's decomposition showed educational differences explaining approximately 35 % of ethnic disparities, with household wealth contributing an additional 20 %. Urban-rural differences explained 15 % of the observed variations.

#### 8.5.2 Western Africa

Nigeria's decomposition showed educational differences accounting for the largest share (30–35 %) of explained variation in the Hausa-Yoruba disparity, followed by household wealth (20–25 %). Child characteristics explained a relatively small proportion (10–15 %). In Burkina Faso, household wealth emerged as the dominant explanatory factor (35–40 %) for the Mossi-Gourmantche comparison, while educational differences explained a smaller proportion (15–20 %). Guinea's analysis revealed that maternal education explained approximately 25 % of ethnic disparities, with household wealth contributing an additional 20 %.

#### 8.5.3 Central and Southern Africa

In Cameroon, educational differences emerged as the primary driver of ethnic disparities, explaining approximately 30 % of observed differences. Household wealth contributions varied substantially across ethnic contrasts (15–25 %). Zambia's decomposition showed household wealth explaining 25–30 % of ethnic disparities, followed by maternal education (20 %). Urban-rural differences contributed an additional 15 % to the explanation of observed disparities.

## 9 Cross-cutting patterns

### 9.1 Temporal stability

Most ethnic disparities showed remarkable stability between surveys. In Kenya, the Luo-Kalenjin disparity remained nearly unchanged (difference in adjusted RR -0·02). Similar stability appeared in Nigeria and Burkina Faso, where ethnic disparities showed minimal temporal variation.

### 9.2 Socioeconomic adjustment effects

The impact of socioeconomic adjustment varied markedly across contexts. While some disparities, like the Luo-Kalenjin in Kenya, showed minimal change after adjustment, others demonstrated substantial attenuation. In Nigeria, the Yoruba-Hausa disparity significantly reduced after adjustment, suggesting strong socioeconomic mediation. Urban-rural differences contributed consistently to ethnic disparities across most countries, though their importance varied. In Uganda and Zambia, geographic factors explained approximately 15 % of ethnic disparities, while in Kenya and Nigeria, their contribution was more modest (10–12 %). Maternal education emerged as a crucial factor across most countries, though its importance varied. Educational differences explained the most significant proportion of ethnic disparities in Nigeria (30–35 %) and Uganda (35 %), while contributing more modestly to Burkina Faso (15–20 %) and Guinea (25 %).

## 10 Discussion

The findings demonstrate that ethnic disparities in under-five mortality across sub-Saharan Africa are driven less by intrinsic cultural traits and more by entrenched structural and institutional inequalities rooted in colonial legacies and administrative geography. Although child, maternal, and household characteristics attenuate some disparities, they persist because deeper historical and geographic determinants exist.

Colonial regimes reinforced interethnic inequalities that persist today since they structured health investments unevenly. British colonial authorities in Nigeria invested disproportionately in the Southern regions compared to the North. This investment shaped contemporary disparities between Hausa and Yoruba children (Mamdani, 1996; Antai, 2011). In Kenya, the Kikuyu benefited disproportionately from early health and education investments, marginalizing groups like the Luo. The findings align with Van Malderen et al.'s (2019) multi-country analysis, which documented persistent ethnic health disparities across 20 sub-Saharan countries, though their study did not examine geographic mediation. Similarly, Quentin et al. (2014) found ethnic disparities in four African countries but attributed these primarily to socioeconomic factors, explaining 27–52 % of inequalities—comparable to our 25–35 % for maternal education.

The sensitivity analysis reveals that administrative geography mediates ethnic disparities because controlling for administrative region reduces ethnic mortality differences by 15.4 % in Ghana and 35.1 % in Nigeria. These findings address the theoretical tension between ethnicity as a cultural construct versus a geographically contingent identity. They show that ethnic disadvantage is not static but mediated by the institutional and spatial contexts in which ethnic groups reside. The more substantial attenuation observed in Nigeria supports the literature on healthcare decentralization. Nigeria's state-led healthcare model is one example of a decentralized system. Policy implementation and resource allocation with service quality show subnational heterogeneity inside these systems (Bossert et al., 2003). The magnitude of Hausa-Yoruba disparities documented (adjusted RR 1.39) is consistent with Adedini et al.'s (2023) findings using 2018 DHS data, though lower than Antai's (2011) earlier estimates (OR 2.14), suggesting potential convergence over time. However, the geographic mediation analysis extends these studies by demonstrating that 35 % of these disparities operate through administrative boundaries rather than ethnic identity per se.

In contrast, Ghana's centralized National Health Insurance Scheme operates under uniform national guidelines, limiting regional variation in access and delivery. Thus, administrative controls substantially reduce ethnic disparities in Nigeria, lending empirical support to Mamdani's (1996) argument that colonial-era administrative structures continue to influence health outcomes through institutional channels. Ghana's narrowing ethnic gaps corroborate Orangi et al.'s (2021) evaluation of free maternity policies, which found differential uptake across ethnic groups initially but convergence over time. This contrasts with persistent disparities documented in neighboring countries despite similar policies (Dzakpasu et al., 2013; Wang et al., 2017; Imo et al., 2022), suggesting implementation quality matters more than policy adoption.

The homeland–diaspora framework adds even further subtlety since it shows that between 25 % and 89 % of those ethnic groups reside outside their customary homelands. Mortality rates differ among co-ethnics in the homeland and diaspora regions. These distinctions question beliefs that ethnicity fits cleanly with geographic place. Brockerhoff and Hewett (2000) find that ethnicity intersects with rural–urban disparities. This intersection shapes child health outcomes in alignment with these findings.

The interaction between ethnicity and administrative location suggests that cultural practices alone cannot explain mortality risk. Healthcare-seeking behavior among Fulani pastoralists varies with local health system structure and the presence of cultural brokers but not ethnicity per se (Hampshire et al., 2009). Cultural norms and the administrative and institutional context interact, reflecting ethnic disparities. Apparent “cultural” behaviors, such as early complementary feeding, often arise when poor health infrastructure is present, so nutrition counseling is limited in regions (Pelto & Armar‐Klemesu, 2011; Hicken et al., 2018). This logic is echoed through our findings: geographic controls cause strong attenuation of ethnic effects, suggesting administrative boundaries proxy service quality and healthcare access variation.

These findings underscore that health policies function within administrative—not ethnic—boundaries: health policies function within administrative—not ethnic—boundaries. Including state or regional controls reduces ethnic disparities substantially, especially in Nigeria, where attenuation averaged 35.1 %. This underscores the risk of ethnic-targeted interventions that ignore subnational geographic heterogeneity in healthcare delivery. Policy design must therefore prioritize administrative geography as ethnic diversity is considered intra-regionally. Okoli et al. (2020) argue that decentralized policies can have negative impacts. Ethnic minorities far outside their customary lands may be marginalized. These findings confirm this concern that health interventions focused on ethnic risk overlook some variation in mortality risk driven by geographic and institutional contexts.

### 10.1 Methodological contributions and limitations

Unlike the Kitagawa–Blinder–Oaxaca decomposition Van Malderen et al. (2019) employed, the approach used attributes path-independently even when explanatory variables like maternal education, household wealth, and geographic location correlate. Given historically embedded marginalization, this is especially important when considering how these factors cluster along ethnic lines. Disparities among non-reference groups may be obscured by customary reference-category approaches (e.g., Victora et al., 2020). For instance, these approaches would mask mortality risk differences that this approach identifies between Hausa and Yoruba children in Nigeria.

This framework assesses ethnicity within administrative boundary interactions, filling a larger publication void. This is of critical importance since it answers so many theoretical questions. Studies that treat ethnicity as a cultural concept overlook the substantial geographic mediation documented. Ethnic disparities attenuate significantly with geographic controls; nevertheless, residual effects persist. These can reflect dimensions like how well healthcare serves, how providers may discriminate, whether cultural brokers are present or absent, and what community-level social capital exists—all of which probably vary along ethnic and geographic lines.

Hampshire et al. (2009) documented how pluralistic medical practices and provider mistrust shape engagement by Fulani communities with health systems, because our findings suggest that apparent ethnic disparities often reflect broader institutional and geographic barriers. Administrative boundaries mediate outcomes, so this supports geographically targeting interventions through culturally capable care and community health worker programs. We should not depend on ethnic targeting by itself.

### 10.2 Implications for health equity policy

Our findings suggest fundamentally reframing ethnic health disparities from cultural to institutional explanations. The substantial geographic mediation we document indicates that interventions addressing administrative and institutional barriers may be more effective than cultural competency approaches alone. Specifically, the more substantial mediation effects in Nigeria's decentralized system suggest that federal health systems require explicit coordination mechanisms to prevent administrative boundaries from becoming barriers to equitable healthcare access. Ghana's more centralized approach appears to reduce such geographic stratification, though at the cost of potentially limited local responsiveness.

### 10.3 Future research directions

The sensitivity analysis opens several critical research questions: Temporal Dynamics: How do ethnic-geographic interactions evolve as internal migration patterns change and administrative boundaries are redrawn? Service Delivery Mechanisms: What aspects of healthcare delivery (infrastructure, staffing, supply chains, cultural competency) drive the geographic mediation effects we observe? Policy Evaluation: How can health system reforms explicitly address the intersection of ethnic identity and administrative residence to prevent geographic disparities from reinforcing ethnic marginalization?

## 11 Conclusion

This analysis demonstrates that ethnic health disparities in sub-Saharan Africa operate through complex interactions between cultural identity, historical marginalization, and contemporary administrative structures. Rather than viewing ethnicity and geography as competing explanations, our findings suggest they are fundamentally intertwined, with administrative boundaries as critical mediators of apparent ethnic health differences.

The substantial geographic mediation we document—particularly in decentralized healthcare systems—highlights the importance of institutional rather than purely cultural approaches to addressing ethnic health disparities. Achieving health equity requires policies that explicitly address the intersection of ethnic identity and administrative residence, ensuring that healthcare delivery systems do not inadvertently reinforce historical patterns of marginalization through contemporary geographic inequities.

## Ethics declarations

The research was based on analyzing anonymized secondary data sources in the DHS public domain. Therefore, no further approval was expected. That being said, the author has gained permission to reuse the data.

## Funding

No funding was received for this study.

## Conflict of interest

The author declares no competing interests.

## Figures and Tables

**Table 3:** Ethnic Disparities in crude and adjusted mortality rates

Ethnicity	% share	crude rate (Survey 1)	Adjusted rate (Survey 1)	crude rate (Survey 2)	Adjusted rate (Survey 2)
**Burkina Faso**					
Mossi	0.516	1	1	1	1
Other	0.095	0.90 (0.76–1.07)	0.92 (0.78–1.09)	0.90 (0.69–1.16)	0.92 (0.72–1.17)
Fulfuldé/Peul	0.081	1.32 (1.10–1.59)	1.20 (1.02–1.42)	1.32 (1.01–1.72)	1.20 (0.94–1.54)
Gourmantche	0.059	1.53 (1.32–1.77)	1.37 (1.18–1.58)	1.53 (1.21–1.92)	1.36 (1.09–1.70)
Gourounsi	0.046	0.87 (0.69–1.09)	0.85 (0.68–1.06)	0.87 (0.63–1.18)	0.85 (0.63–1.14)
Bissa	0.043	0.60 (0.49–0.73)	0.61 (0.50–0.74)	0.59 (0.45–0.79)	0.60 (0.46–0.80)
Senoufo	0.042	1.52 (1.31–1.76)	1.52 (1.33–1.74)	1.51 (1.20–1.90)	1.52 (1.22–1.88)
Lobi	0.036	1.63 (1.33–1.99)	1.53 (1.26–1.87)	1.62 (1.22–2.16)	1.53 (1.16–2.02)
Bobo	0.033	0.89 (0.71–1.12)	0.91 (0.72–1.15)	0.89 (0.65–1.21	0.91 (0.67–1.24)
Dagara	0.033	1.18 (0.96–1.44)	1.15 (0.94–1.41)	1.17 (0.88–1.57	1.15 (0.87–1.52)
Touareg/Bella	0.009	1.56 (1.30–1.87)	1.35 (1.11–1.64)	1.55 (1.19–2.02)	1.35 (1.03–1.77)
Dioula	0.007	1.19 (0.84–1.68)	1.31 (0.96–1.78)	1.18 (0.77–1.82)	1.31 (0.89–1.92
**Cameroon**					
Arab-Choa	0.352	1	1	1	1
Other	0.303	0.75 (0.66–0.86)	0.89 (0.78–1.01)	0.74 (0.58–0.95)	0.88 (0.69–1.11)
Biu-Mandara	0.237	0.69 (0.61–0.78)	1.04 (0.92–1.18)	0.68 (0.54–0.87)	1.03 (0.81–1.30)
Grassfields	0.040	0.35 (0.26–0.48)	0.56 (0.42–0.76)	0.35 (0.22–0.53)	0.56 (0.37–0.84)
Kako/Meka/Pygmé	0.024	1.08 (0.78–1.48)	1.28 (0.95–1.71)	1.06 (0.69–1.65)	1.26 (0.84–1.89)
Bantoïde Southwest	0.013	0.95 (0.63–1.42)	0.81 (0.53–1.23)	0.94 (0.55–1.58)	0.80 (0.47–1.36)
Adamaoua-Oubangui	0.012	2.24 (1.66–3.02)	1.82 (1.39–2.39)	2.22 (1.46–3.36)	1.80 (1.23–2.64)
Bamilike/Bamoun	0.010	0.63 (0.30–1.33)	0.87 (0.42–1.80)	0.62 (0.26–1.48)	0.86 (0.38–1.99)
Beti/Bassa/Mbam	0.009	0.91 (0.58–1.42)	1.26 (0.79–2.00)	0.90 (0.51–1.58)	1.25 (0.70–2.21)
**Congo, DR**					
Kasai, Katanga, Tanganika	0.290	1	1	1	1
Basele-K, Man. and Kivu	0.201	1.02 (0.83–1.26)	0.99 (0.81–1.19)	1.08 (0.74–1.58)	1.03 (0.73–1.46)
Bas-Kasai and Kwilu-Kwngo	0.138	1.00 (0.70–1.42)	1.00 (0.73–1.35)	1.06 (0.62–1.79)	1.04 (0.66–1.66)
Ubangi and Itimbiri	0.101	1.18 (0.83–1.70)	1.07 (0.76–1.50)	1.25 (0.73–2.13)	1.12 (0.68–1.84)
Cuvette central	0.093	0.75 (0.58–0.96)	0.72 (0.57–0.91)	0.79 (0.52–1.21)	0.76 (0.51–1.12)
Bakongo North and South	0.087	1.04 (0.83–1.30)	1.24 (1.02–1.51)	1.09 (0.73–1.63)	1.30 (0.92–1.85)
Uele Lake Albert	0.072	1.40 (1.04–1.88)	1.21 (0.90–1.62)	1.48 (0.92–2.36)	1.27 (0.81–1.99)
Lunda	0.010	1.00 (0.73–1.35	0.92 (0.61–1.39)	1.05 (0.65–1.70)	0.97 (0.55–1.71)
Others	0.008	1.51 (1.11–2.05)	1.52 (1.12–2.07)	1.59 (0.98–2.58)	1.60 (1.01–2.54)
**Gabon**					
Other Gabon	0.239	1	1	1	1
Shira-Punu	0.207	0.95 (0.63–1.42)	1.03 (0.69–1.55)	0.89 (0.41–1.92)	0.97 (0.44–2.12)
Fang	0.182	1.22 (0.86–1.72)	1.38 (0.96–1.98)	1.14 (0.56–2.34)	1.30 (0.62–2.70)
Other	0.162	0.79 (0.56–1.13)	0.89 (0.61–1.29)	0.74 (0.36–1.53)	0.83 (0.40–1.76)
Nzebi-Duma	0.124	0.98 (0.64–1.50)	1.04 (0.67–1.59)	1.03 (0.67–1.59)	0.97 (0.44–2.17)
Mbede-Teke	0.052	0.60 (0.35–1.01)	0.63 (0.37–1.08)	0.56 (0.23–1.37)	0.59 (0.24–1.48)
Myene	0.034	0.32 (0.12–0.84)	0.39 (0.15–1.03)	0.30 (0.08–1.14)	0.37 (0.10–1.41)
**Ghana**					
Akan	0.342	1	1	1	1
Mole-Dagbani	0.273	1.28 (1.04–1.57)	0.97 (0.78–1.21)	1.28 (0.89–1.86)	0.97 (0.67–1.41)
Gurma	0.110	1.57 (1.28–1.93)	1.11 (0.88–1.40)	1.58 (1.10–2.28)	1.11 (0.75–1.63)
Ewe	0.104	0.96 (0.76–1.22)	0.88 (0.70–1.11)	0.97 (0.65–1.44)	0.88 (0.60–1.30)
Grusi	0.048	1.14 (0.77–1.70)	0.92 (0.62–1.38)	1.15 (0.66–2.01)	0.92 (0.53–1.62)
Ga/Dangme	0.043	0.88 (0.62–1.24)	0.85 (0.61–1.19)	0.88 (0.53–1.46)	0.85 (0.52–1.39)
Guan	0.041	1.51 (1.07–2.13)	1.43 (1.03–1.99)	1.52 (0.92–2.51)	1.43 (0.88–2.33)
Mande	0.027	1.15 (0.66–2.01)	0.89 (0.51–1.54)	1.16 (0.56–2.38)	0.89 (0.44–1.80)
Other	0.012	0.80 (0.41–1.56)	0.67 (0.35–1.29)	0.81 (0.35–1.84)	0.67 (0.30–1.50)
**Guinea**					
Peulh	0.411	1	1	1	1
Malinké	0.312	1.19 (1.03–1.38)	1.20 (1.05–1.37)	1.23 (0.92–1.63)	1.22 (0.94–1.58)
Soussou	0.168	0.88 (0.72–1.07)	1.00 (0.83–1.21)	0.90 (0.65–1.26)	1.02 (0.74–1.40)
Kissi	0.048	1.17 (0.93–1.48)	1.10 (0.88–1.39)	1.20 (0.83–1.74)	1.12 (0.79–1.60)
Guerzé	0.040	0.70 (0.51–0.96)	0.70 (0.51–0.95)	0.72 (0.45–1.13)	0.71 (0.46–1.09)
Other	0.012	1.28 (0.94–1.74)	1.36 (0.99–1.88)	1.31 (0.84–2.05)	1.38 (0.89–2.17)
Toma	0.009	0.43 (0.19–0.97)	0.42 (0.19–0.95)	0.44 (0.17–1.15)	0.43 (0.17–1.09)
**Kenya**					
Kalenjin	0.178	1	1	1	1
Luo	0.132	2.17 (1.85–2.54)	2.15 (1.83–2.51)	2.15 (1.55–2.99)	2.13 (1.54–2.95)
Kikuyu	0.122	1.14 (0.94–1.39)	1.29 (1.06–1.56)	1.14 (0.79–1.63)	1.28 (0.89–1.83)
Luhya	0.118	1.51 (1.27–1.81)	1.56 (1.31–1.85)	1.50 (1.06–2.12)	1.55 (1.10–2.18)
Somali	0.088	1.33 (0.83–2.14)	1.49 (0.93–2.38)	1.32 (0.69–2.52)	1.48 (0.78–2.79)
Kamba	0.079	0.90 (0.73–1.12)	0.94 (0.76–1.17)	0.90 (0.61–1.31)	0.94 (0.64–1.37)
Other	0.075	1.48 (1.20–1.83)	1.44 (1.15–1.79)	1.47 (1.01–2.15)	1.43 (0.97–2.10)
Mijikenda/Swahili	0.054	1.18 (0.95–1.47)	1.14 (0.91–1.42)	1.18 (0.80–1.73)	1.13 (0.77–1.67)
Meru	0.053	1.18 (0.92–1.51)	1.23 (0.96–1.56)	1.18 (0.78–1.78)	1.22 (0.81–1.83)
Kisii	0.053	0.79 (0.61–1.03)	0.85 (0.65–1.09)	0.78 (0.51–1.21)	0.84 (0.55–1.28)
Maasai	0.027	0.68 (0.47–0.98)	0.64 (0.44–0.93)	0.68 (0.40–1.16)	0.64 (0.37–1.09)
Taita/Taveta	0.012	1.33 (0.83–2.14)	1.49 (0.93–2.38)	1.32 (0.69–2.52)	1.48 (0.78–2.79)
Embu	0.009	1.70 (0.92–3.13)	1.78 (0.97–3.25)	1.69 (0.78–3.69)	1.77 (0.82–3.81)
**Nigeria**					
Hausa	0.324	1	1	1	1
Other	0.263	0.58 (0.53–0.63)	0.66 (0.60–0.71)	0.57 (0.49–0.67)	0.65 (0.57–0.75)
Igbo	0.120	0.49 (0.43–0.56)	0.76 (0.68–0.86)	0.49 (0.40–0.60)	0.76 (0.64–0.91)
Yoruba	0.105	0.41 (0.36–0.46)	0.72 (0.64–0.82)	0.40 (0.33–0.49)	0.72 (0.60–0.87)
Fulani	0.091	0.80 (0.71–0.89)	0.75 (0.67–0.83)	0.80 (0.66–0.96)	0.75 (0.63–0.88)
Ijaw/Izon	0.031	0.50 (0.40–0.62)	0.66 (0.55–0.81)	0.49 (0.37–0.66)	0.66 (0.51–0.85)
Kanuri/Beriberi	0.020	0.58 (0.47–0.72)	0.63 (0.52–0.77)	0.58 (0.43–0.77)	0.63 (0.49–0.81)
Tiv	0.018	0.48 (0.39–0.58)	0.49 (0.41–0.59)	0.47 (0.36–0.62)	0.49 (0.39–0.63)
Ibibio	0.015	0.34 (0.25–0.47)	0.50 (0.36–0.71)	0.34 (0.23–0.51)	0.50 (0.34–0.75)
Igala	0.009	0.49 (0.35–0.67)	0.61 (0.43–0.85)	0.48 (0.33–0.72)	0.61 (0.41–0.90)
Ekoi	0.003	0.66 (0.31–1.42)	0.84 (0.41–1.69)	0.66 (0.28–1.52)	0.84 (0.39–1.79)
**Senegal**					
Poular	0.349	1	1	1	1
Wolof	0.332	0.82 (0.67–1.02)	0.91 (0.74–1.11)	0.87 (0.57–1.34)	0.96 (0.64–1.43)
Serer	0.118	0.90 (0.66–1.22)	0.95 (0.71–1.29)	0.95 (0.56–1.60)	1.01 (0.61–1.66)
Mandingue/Socé	0.084	1.25 (0.85–1.83)	1.44 (1.00–2.09)	1.32 (0.73–2.40)	1.52 (0.86–2.70)
Diola	0.038	0.57 (0.29–1.15)	0.73 (0.37–1.46)	0.61 (0.25–1.50)	0.77 (0.32–1.89)
Not Senegalese	0.032	1.04 (0.69–1.56)	1.19 (0.83–1.72)	1.10 (0.60–2.04)	1.26 (0.72–2.22)
Other ethnicity	0.024	1.46 (0.72–2.94)	1.66 (0.85–3.25)	1.55 (0.62–3.85)	1.75 (0.73–4.19)
Soninke	0.024	0.40 (0.22–0.73)	0.54 (0.33–0.90)	0.43 (0.19–0.96)	0.58 (0.28–1.16)
Mende	0.353	1	1	1	1
**Sierra Leone**					
Temne	0.316	0.85 (0.76–0.96)	0.87 (0.78–0.98)	0.83 (0.68–1.03)	0.85 (0.70–1.05)
Limba	0.071	0.63 (0.52–0.78)	0.68 (0.55–0.83)	0.62 (0.46–0.84)	0.67 (0.50–0.89)
Other Sierra Leone	0.056	0.76 (0.62–0.93)	0.77 (0.63–0.93)	0.75 (0.56–1.00)	0.75 (0.57–1.00)
Koranko/Korankoh	0.049	0.89 (0.72–1.10)	0.87 (0.71–1.07)	0.87 (0.65–1.18)	0.86 (0.64–1.15)
Kono	0.048	0.81 (0.68–0.96)	0.82 (0.69–0.98)	0.80 (0.61–1.04)	0.81 (0.62–1.05)
Fullah	0.032	0.52 (0.40–0.68)	0.58 (0.45–0.75)	0.51 (0.36–0.74)	0.57 (0.40–0.81)
Mandingo	0.025	0.84 (0.67–1.06)	0.93 (0.75–1.17)	0.83 (0.60–1.14)	0.92 (0.67–1.26)
Sherbro	0.023	0.92 (0.72–1.18)	0.91 (0.71–1.16)	0.90 (0.64–1.28)	0.89 (0.64–1.24)
Loko	0.020	0.66 (0.50–0.88)	0.70 (0.53–0.92)	0.65 (0.44–0.94)	0.69 (0.48–0.98)
Creole	0.004	0.36 (0.19–0.69)	0.47 (0.24–0.89)	0.35 (0.17–0.75)	0.46 (0.22–0.96)
Other Foreign	0.004	0.66 (0.38–1.17)	0.71 (0.41–1.25)	0.65 (0.34–1.26)	0.70 (0.36–1.34)
**Uganda**					
Baganda	0.181	1	1	1	1
Langi	0.112	1.09 (0.89–1.35)	0.94 (0.76–1.16)	1.07 (0.74–1.57)	0.92 (0.64–1.34)
Basoga	0.096	1.04 (0.81–1.32)	0.95 (0.74–1.22)	1.02 (0.67–1.54)	0.94 (0.62–1.41)
Iteso	0.088	0.87 (0.68–1.10)	0.70 (0.56–0.88)	0.85 (0.57–1.28)	0.69 (0.47-1.02)
Bakiga	0.087	1.27 (1.01–1.59)	1.13 (0.91–1.40)	1.24 (0.84–1.85)	1.11 (0.76–1.62)
Banyankole	0.086	1.41 (1.14–1.76)	1.30 (1.06–1.60)	1.39 (0.94–2.04)	1.28 (0.89–1.86)
Other	0.066	1.26 (0.97–1.63)	1.08 (0.84–1.39)	1.23 (0.80–1.89)	1.06 (0.70–1.61)
Bagisu	0.056	0.91 (0.72–1.15)	0.80 (0.63–1.00)	0.90 (0.60–1.34)	0.78 (0.53–1.16)
Lugbara	0.043	1.16 (0.89–1.50)	0.92 (0.72–1.19	1.14 (0.74–1.75)	0.91 (0.60–1.38)
Bakonzo	0.031	1.04 (0.72–1.50)	0.97 (0.68–1.39)	1.02 (0.60–1.74)	0.96 (0.57–1.61)
Bafumbira	0.028	1.71 (1.23–2.37)	1.37 (1.00–1.88)	1.67 (1.02–2.75)	1.35 (0.84–2.18)
Banyarwanda	0.025	1.11 (0.78–1.58)	1.04 (0.75–1.46)	1.09 (0.65–1.84)	1.03 (0.62–1.69)
Banyoro	0.022	0.76 (0.43–1.33)	0.75 (0.43–1.32)	0.74 (0.36–1.54)	0.74 (0.36–1.52)
Bagwere	0.018	0.73 (0.54–1.00)	0.64 (0.48–0.85)	0.72 (0.45–1.16)	0.63 (0.41–0.98)
Acholi	0.018	1.28 (0.82–2.00)	0.97 (0.61–1.53)	1.26 (0.68–2.32)	0.95 (0.51–1.78)
Batoro	0.017	0.96 (0.64–1.46)	0.95 (0.62–1.45)	0.95 (0.53–1.70)	0.93 (0.52–1.68)
Madi	0.012	0.87 (0.55–1.39)	0.73 (0.47–1.13)	0.86 (0.45–1.62)	0.72 (0.39–1.31)
Karimojong	0.012	1.42 (0.89–2.28)	0.90 (0.57–1.41)	1.40 (0.74–2.65)	0.88 (0.48–1.63)
Alur	0.002	1.50 (0.96–2.35)	1.12 (0.74–1.70)	1.47 (0.80–2.73)	1.10 (0.62–1.97)
**Zambia**					
Bemba	0.312	1	1	1	1
Other	0.302	1.03 (0.90–1.16)	1.03 (0.91–1.16)	1.03 (0.79–1.33)	1.03 (0.80–1.32)
Tonga	0.126	0.78 (0.63–0.95)	0.82 (0.67–1.01)	0.78 (0.56–1.09)	0.82 (0.59–1.15)
Nyanja	0.090	0.91 (0.75–1.09)	0.90 (0.74–1.08)	0.91 (0.66–1.25)	0.90 (0.65–1.23)
Lozi	0.065	1.04 (0.80–1.34)	1.01 (0.79–1.30)	1.04 (0.70–1.53)	1.01 (0.69–1.48)
Lunda	0.038	0.61 (0.46–0.80)	0.61 (0.47-0.80	0.61 (0.40–0.91)	0.61 (0.41–0.91)
Kaonde	0.036	0.70 (0.51–0.95)	0.72 (0.53–0.99)	0.70 (0.45–1.08)	0.72 (0.47–1.12)
Luvale	0.031	0.70 (0.45–1.09)	0.68 (0.44–1.07)	0.71 (0.40–1.25)	0.68 (0.39–1.21)

**Table:** 

Contrast	survey year	estimate	SE	z.ratio	p.value
Burkina Faso					
Mossi - Gourmantche	2010	−0.312	0.073	−4.284	0.001
Mossi - Bissa	2010	0.501	0.102	4.891	0.000
Mossi - Senoufo	2010	−0.42	0.069	−6.106	0.000
Mossi - Lobi	2010	−0.427	0.101	−4.218	0.001
Other - Gourmantche	2010	−0.394	0.111	−3.551	0.020
Other - Bissa	2010	0.419	0.128	3.272	0.049
Other - Senoufo	2010	−0.502	0.097	−5.152	0.000
Other - Lobi	2010	−0.509	0.129	−3.939	0.005
Fulfuldé/Peul - Bissa	2010	0.686	0.128	5.350	0.000
Gourmantche - Gourounsi	2010	0.476	0.127	3.748	0.010
Gourmantche - Bissa	2010	0.813	0.121	6.702	0.000
Gourounsi - Senoufo	2010	−0.583	0.125	−4.677	0.000
Gourounsi - Lobi	2010	−0.591	0.146	−4.037	0.003
Bissa - Senoufo	2010	−0.921	0.119	−7.764	0.000
Bissa - Lobi	2010	−0.928	0.140	−6.650	0.000
Bissa - Dagara	2010	−0.643	0.140	−4.577	0.000
Bissa - (Touareg/Bella)	2010	−0.803	0.140	−5.743	0.000
Bissa - Dioula	2010	−0.771	0.186	−4.154	0.002
Senoufo - Bobo	2010	0.512	0.129	3.977	0.004
Lobi - Bobo	2010	0.52	0.150	3.475	0.026
Mossi - Gourmantche	2021	−0.306	0.080	−3.837	0.007
Mossi - Bissa	2021	0.473	0.105	4.503	0.000
Mossi - Senoufo	2021	−0.414	0.072	−5.755	0.000
Mossi - Lobi	2021	−0.406	0.104	−3.908	0.005
Other - Gourmantche	2021	−0.406	0.116	−3.507	0.023
Other - Senoufo	2021	−0.514	0.098	−5.232	0.000
Other - Lobi	2021	−0.506	0.130	−3.886	0.006
Fulfuldé/Peul - Bissa	2021	0.676	0.135	5.019	0.000
Gourmantche - Gourounsi	2021	0.462	0.137	3.376	0.036
Gourmantche - Bissa	2021	0.779	0.127	6.155	0.000
Gourounsi - Senoufo	2021	−0.569	0.133	−4.295	0.001
Gourounsi - Lobi	2021	−0.561	0.154	−3.648	0.014
Bissa - Senoufo	2021	−0.887	0.122	−7.292	0.000
Bissa - Lobi	2021	−0.879	0.142	−6.193	0.000
Bissa - Dagara	2021	−0.608	0.139	−4.356	0.001
Bissa - (Touareg/Bella)	2021	−0.777	0.141	−5.503	0.000
Bissa - Dioula	2021	−0.685	0.205	−3.349	0.039
Senoufo - Bobo	2021	0.455	0.131	3.479	0.025
Cameroon					
(Arab-Choa) - Grassfields	2011	0.570954	0.153206	3.727	0.006
(Arab-Choa) - (Adamaoua-Oubangui)	2011	−0.60039	0.137427	−4.369	0.000
Other - (Adamaoua-Oubangui)	2011	−0.71949	0.144171	−4.991	0.000
(Biu-Mandara) - Grassfields	2011	0.610261	0.152256	4.008	0.000
(Biu-Mandara) - (Adamaoua-Oubangui)	2011	−0.56109	0.148394	−3.781	0.005
Grassfields - (Kako/Meka/Pygmé)	2011	−0.81415	0.202939	−4.012	0.002
Grassfields - (Adamaoua-Oubangui)	2011	−1.17135	0.201928	−5.801	0.000
(Bantoïde South-West) - (Adamaoua-Oubangui)	2011	−0.81644	0.246446	−3.313	0.026
(Arab-Choa) - Grassfields	2018	0.568813	0.155771	3.652	0.008
(Arab-Choa) - (Adamaoua-Oubangui)	2018	−0.61133	0.138908	−4.401	0.000
Other - (Adamaoua-Oubangui)	2018	−0.72156	0.144498	−4.994	0.000
(Biu-Mandara) - Grassfields	2018	0.574655	0.154513	3.719	0.006
(Biu-Mandara) - (Adamaoua-Oubangui)	2018	−0.60549	0.151393	−3.999	0.0021
Grassfields - (Kako/Meka/Pygmé)	2018	−0.82294	0.202742	−4.059	0.0016
Grassfields - (Adamaoua-Oubangui)	2018	−1.18014	0.201946	−5.844	0.000
(Bantoïde South-West) - (Adamaoua-Oubangui)	2018	−0.81644	0.246335	−3.314	0.0258
Congo, DR					
Cuvette Central - Bakongo North and South	2007	−0.54235	0.138595	−3.913	0.0029
Cuvette central - Others	2007	−0.74648	0.195703	−3.814	0.0043
Kasai, Katanga, Tanganika - Cuvette central	2013	0.476648	0.138519	3.441	0.0169
Cuvette Central - Bakongo North and South	2013	−0.54608	0.154641	−3.531	0.0123
Cuvette central - Others	2013	−0.58791	0.184792	−3.181	0.0393
Gabon					
Fang - Other	2012	0.43	0.142	3.026	0.04
Ghana					
Akan - Gurma	2014	−0.473	0.112	−4.213	0.001
Gurma - Ewe	2014	0.481	0.141	3.409	0.019
Akan - Gurma	2022	−0.525	0.093	−5.649	0.000
Gurma - Ewe	2022	0.570	0.126	4.529	0.000
Gurma - (Ga/Dangme)	2022	0.690	0.19	3.639	0.008
Guinea					
Malinké - Soussou	2012	0.306	0.100	3.065	0.035
Malinké - Guerzé	2012	0.538	0.168	3.198	0.023
Malinké - Soussou	2018	0.320	0.094	3.396	0.012
Malinké - Guerzé	2018	0.533	0.153	3.489	0.009
Kenya					
Kalenjin - Luo	2014	−0.773	0.082	−9.48	0.000
Kalenjin - Luhya	2014	−0.413	0.091	−4.556	0.000
Kalenjin - Other	2014	−0.395	0.107	−3.674	0.015
Luo - Kikuyu	2014	0.638	0.094	6.778	0.000
Luo - Luhya	2014	0.359	0.087	4.114	0.003
Luo - Somali	2014	0.680	0.106	6.395	0.000
Luo - Kamba	2014	0.877	0.103	8.518	0.000
Luo - Other	2014	0.378	0.101	3.747	0.011
Luo - (Mijikenda/Swahili)	2014	0.605	0.107	5.629	0.000
Luo - Meru	2014	0.604	0.122	4.939	0.000
Luo - Kisii	2014	1.009	0.128	7.862	0.000
Luo - Maasai	2014	1.158	0.185	6.275	0.000
Luhya - Kamba	2014	0.517	0.115	4.492	0.001
Luhya - Kisii	2014	0.649	0.138	4.714	0.000
Luhya - Maasai	2014	0.799	0.191	4.192	0.002
Kamba - Other	2014	−0.499	0.127	−3.922	0.006
Other - Kisii	2014	0.631	0.154	4.099	0.003
Other - Maasai	2014	0.780	0.198	3.933	0.006
Kalenjin - Luo	2022	−0.754	0.080	−9.453	0.000
Kalenjin - Luhya	2022	−0.417	0.090	−4.618	0.000
Kalenjin - Other	2022	−0.403	0.107	−3.774	0.010
Luo - Kikuyu	2022	0.575	0.091	6.323	0.000
Luo - Luhya	2022	0.336	0.087	3.870	0.007
Luo - Somali	2022	0.542	0.123	4.394	0.001
Luo - Kamba	2022	0.887	0.102	8.674	0.000
Luo - Other	2022	0.351	0.100	3.508	0.027
Luo - (Mijikenda/Swahili)	2022	0.592	0.108	5.486	0.000
Luo - Meru	2022	0.570	0.123	4.614	0.000
Luo - Kisii	2022	1.045	0.126	8.325	0.000
Luo - Maasai	2022	1.157	0.187	6.192	0.000
Luhya - Kamba	2022	0.551	0.114	4.818	0.000
Luhya - Kisii	2022	0.709	0.135	5.253	0.000
Luhya - Maasai	2022	0.821	0.193	4.260	0.001
Kamba - Other	2022	−0.536	0.127	−4.239	0.002
Other - Kisii	2022	0.694	0.151	4.601	0.000
Other - Maasai	2022	0.807	0.200	4.026	0.004
Nigeria					
Hausa - Other	2013	0.553	0.045	12.325	0.000
Hausa - Igbo	2013	0.711	0.066	10.777	0.000
Hausa - Yoruba	2013	0.904	0.066	13.692	0.000
Hausa - Fulani	2013	0.226	0.058	3.872	0.005
Hausa - (Ijaw/Izon)	2013	0.702	0.113	6.237	0.000
Hausa - (Kanuri/Beriberi)	2013	0.546	0.110	4.963	0.000
Hausa - Tiv	2013	0.741	0.102	7.284	0.000
Hausa - Ibibio	2013	1.067	0.164	6.498	0.000
Hausa - Igala	2013	0.722	0.164	4.41	0.001
Other - Yoruba	2013	0.351	0.069	5.057	0.000
Other - Fulani	2013	−0.327	0.061	−5.36	0.000
Igbo - Fulani	2013	−0.485	0.079	−6.127	0.000
Yoruba - Fulani	2013	−0.678	0.080	−8.524	0.000
Fulani - (Ijaw/Izon)	2013	0.477	0.121	3.926	0.004
Fulani - Tiv	2013	0.515	0.111	4.643	0.000
Fulani - Ibibio	2013	0.841	0.169	4.988	0.000
Hausa - Other	2018	0.588	0.043	13.567	0.000
Hausa - Igbo	2018	0.761	0.063	12.080	0.000
Hausa - Yoruba	2018	1.027	0.061	16.754	0.000
Hausa - Fulani	2018	0.226	0.058	3.89	0.005
Hausa - (Ijaw/Izon)	2018	0.983	0.114	8.637	0.000
Hausa - (Kanuri/Beriberi)	2018	0.546	0.110	4.963	0.000
Hausa - Tiv	2018	0.735	0.101	7.300	0.000
Hausa - Ibibio	2018	0.909	0.110	8.283	0.000
Hausa - Igala	2018	0.742	0.159	4.677	0.000
Other - Yoruba	2018	0.439	0.064	6.865	0.000
Other - Fulani	2018	−0.362	0.06	−6.029	0.000
Other - (Ijaw/Izon)	2018	0.395	0.116	3.408	0.027
Igbo - Yoruba	2018	0.265	0.077	3.43	0.025
Igbo - Fulani	2018	−0.535	0.077	−6.963	0.000
Yoruba - Fulani	2018	−0.800	0.076	−10.576	0.000
Yoruba - (Kanuri/Beriberi)	2018	−0.480	0.120	−3.991	0.003
Fulani - (Ijaw/Izon)	2018	0.757	0.123	6.174	0.000
Fulani - Tiv	2018	0.509	0.110	4.623	0.000
Fulani - Ibibio	2018	0.683	0.118	5.809	0.000
Senegal					
(Mandingue/Socé) - Soninke	2013	1.132	0.343	3.301	0.022
Other ethnicity - Soninke	2013	1.287	0.421	3.061	0.046
Poular - Soninke	2019	1.046	0.288	3.627	0.007
(Mandingue/Socé) - Soninke	2019	1.262	0.316	3.989	0.002
Sierra Leone					
Mende - Limba	2013	0.459	0.104	4.400	0.001
Mende - Fullah	2013	0.653	0.138	4.735	0.000
Temne - Fullah	2013	0.490	0.140	3.492	0.024
Mende - Limba	2019	0.437	0.094	4.659	0.000
Mende - Fullah	2019	0.650	0.132	4.921	0.000
Temne - Fullah	2019	0.471	0.135	3.494	0.024
Fullah - Sherbro	2019	−0.569	0.171	−3.324	0.042
Uganda					
Iteso - Banyankole	2011	−0.490	0.116	−4.231	0.003
Iteso - Bafumbira	2011	−0.678	0.171	−3.958	0.010
Banyankole - Bagisu	2011	0.437	0.111	3.948	0.011
Banyankole - Bagwere	2011	0.655	0.153	4.28	0.003
Bagisu - Bafumbira	2011	−0.625	0.172	−3.634	0.034
Bafumbira - Bagwere	2011	0.843	0.204	4.142	0.005
Iteso - Bakiga	2016	−0.438	0.099	−4.402	0.002
Iteso - Banyankole	2016	−0.444	0.099	−4.500	0.001
Iteso - Bafumbira	2016	−0.642	0.154	−4.157	0.005
Banyankole - Bagisu	2016	0.387	0.107	3.623	0.035
Bagisu - Bafumbira	2016	−0.585	0.164	−3.566	0.043
Zambia					
Bemba - Lunda	2013	0.500	0.141	3.549	0.009
Other - Lunda	2013	0.525	0.139	3.782	0.004
Bemba - Lunda	2019	0.533	0.157	3.391	0.016
Other - Lunda	2019	0.566	0.157	3.61	0.007

## Data Availability

The microdata used in this study are publicly available from the Demographic and Health Surveys (DHS) Program. All DHS survey files can be obtained at no cost after free registration via the DHS data repository (https://dhsprogram.com/data/). Researchers interested in reproducing the analyses can only access and download the relevant country-year datasets if they have previously registered with the repository. No additional permissions were required.

## Appendix 3 Sensitivity Analyses

In Ghana, relative to the Akan group, Mole-Dagbani (RR = 1.46, 95 % CI: 1.19–1.79, p < 0.001), Gurma (RR = 1.88, 95 % CI: 1.52–2.33, p < 0.001), and also Guan (RR = 1.64, 95 % CI: 1.02–2.64, p = 0.043) exhibited notably higher under-five mortality risks, though other groups showed no meaningful differences. In Nigeria, Hausa (RR = 2.19, 95 % CI: 0.79–6.03) and Fulani (RR = 1.69, 95 % CI: 0.61–4.69) groups also showed greater risks than the Igbo. However, confidence intervals were wide, lacking statistical significance.

Regional adjustments then reduced ethnic disparities within the two countries. Mole-Dagbani with Grusi plus Gurma beside Mande groups saw an ethnic reduction within Ghana. This reduction ranged in value from around 14.8 % to almost 16.4 %. Fulani at 41.5 %, Hausa at 39.8 %, Yoruba at 35.1 %, and Tiv at 33.0 % showed the largest attenuations in Nigeria. Regional variations can mediate large parts of ethnic disparities in decentralized places, as indicated by these observations.

The study did notice impressive group-specific heterogeneity while one resided inside one's ethnic homeland, which was not considerably associated with overall mortality within Ghana (RR = 1.19, 95 % CI: 0.95–1.48). For example, Guan homeland Mothers had a more negligible mortality risk (RR = 0.54, 95 % CI: 0.26–1.12), but Mande mothers risked nearly zero owing to small numbers (RR ≈ 0.00, p < 0.001). A lower risk was connected to Kanuri/Beriberi homeland residence in Nigeria (RR = 0.51, 95 % CI: 0.34–0.75, p = 0.001), yet other groups lacked strong effects.

Homeland–diaspora analysis revealed that 13.5 % (Guan) to 89.7 % (Ibibio) of each group lived inside their customary homelands. Over 60 % of Fulani and Hausa children resided outside their customary territories.

Interaction and homeland models founded upon the AIC improved the model's general fit compared to the baseline, primarily for Ghana. Geographic with migration-sensitive ethnic indicators improve conventional models' explanatory power.
